# Sperm-associated antigen 11A is expressed exclusively in the principal cells of the mouse caput epididymis in an androgen-dependent manner

**DOI:** 10.1186/1477-7827-11-59

**Published:** 2013-07-01

**Authors:** Dwi A Pujianto, Evelyn Loanda, Puji Sari, Yurnadi H Midoen, Purnomo Soeharso

**Affiliations:** 1 Department of Biology, Faculty of Medicine, University of Indonesia, Jl. Salemba Raya 6, Jakarta 10430, Indonesia; 2 Master Program for Biomedical Sciences, Faculty of Medicine, University of Indonesia, Jl. Salemba Raya 6, Jakarta 10430, Indonesia; 3 Department of Biochemistry, Faculty of Medicine, Atma Jaya Catholic University, Jl. Pluit Raya 2, Jakarta 14440, Indonesia

**Keywords:** *Spag11a*, Epididymis, Androgen, Secretory protein, Principal cells

## Abstract

**Background:**

Epididymal sperm maturation occurs via interactions between sperm and proteins secreted by the epididymal epithelium. Although this is an important process, the genes that encode the involved proteins remain largely uncharacterized. Previous studies have demonstrated that the genes involved in sperm maturation are regulated by androgen. *Spag11a* is an epididymal gene that is influenced by androgen. However, little is known about the putative role of this gene in the sperm maturation process. The objective of this study was to characterize *Spag11a* in the mouse epididymis.

**Methods:**

*In silico* analyses were performed to predict signal peptides and functional domains. *Spag11a* expression was measured by quantitative real-time RT-PCR. Western blots and immunocytochemistry were performed to determine protein expression.

**Results:**

SPAG11A is a member of the beta defensin protein family and constitutes a secretory protein. *Spag11a* was expressed exclusively in the epididymis. Moreover, it exhibited region-specific expression in the caput, which is typical for genes that are involved in creating a suitable microenvironment for sperm maturation. Mouse *Spag11a* was regulated by androgen. A significant decrease of *Spag11a* expression was observed at third day following a gonadectomy (P < 0.001). Interestingly, testosterone replacement therapy was able to maintain the expression almost at the normal level, indicating a dependency on androgen. Besides androgen, testicular factors influenced *Spag11a* expression in a different way. This was revealed by efferent duct ligation in which *Spag11a* was transiently up-regulated at the third day following the ligation before returning to the normal level at day 5. *Spag11a* regional expression was also observed at protein level detected by western immunoblotting which revealed a clear band in the caput but not in other regions. The prediction that SPAG11A is a secretory protein was confirmed by immunocytochemical analyses indicating cell-specific expression mainly in the caput principal cells and detection of the protein in epididymal luminal fluid and spermatozoa.

**Conclusions:**

Based on the characteristics of *Spag11a*, it is likely that this gene has a specific role in epididymal sperm maturation. Further studies using functional assays are necessary to confirm this finding.

## Background

Spermatozoa are immotile as they leave the testis and do not have the ability to fertilize an oocyte. To gain the capacity to fertilize, they must undergo a maturation process in the epididymis. This process occurs via interactions between the sperm and proteins secreted by the epididymal epithelium that result in biochemical and physiological changes to the sperm membrane [1-3]. The changes in the sperm membrane include modification or relocalization of pre-existing proteins or the acquisition of new proteins synthesized by the epididymal epithelium.

The mouse epididymis is divided into four distinct regions based on cellular morphology: the initial segment, the caput, the corpus and the cauda [4]. Each region creates its own microenvironment in which the epithelial cells secrete proteins in a highly regulated and regionalized manner so that spermatozoa encounter luminal proteins in a specific sequence [5,6]. This is illustrated by the region-specific expression of epididymal genes that encode several classes of proteins, such as proteases [7], protease inhibitors [8,9], ion transporters [10] and beta defensins [10-12]. In addition to exhibiting highly regionalized expression within the epididymis, such proteins also exhibit tissue specificity; some are expressed only in the epididymis such as lipocalin 5 [13], whereas others are expressed predominantly in the epididymis but also in a few other tissues such as cystatin 8 [8], suggesting that these proteins have a specific role in this organ. Furthermore, there are several epithelial cell types in the ductus epididymis, and several epididymal genes exhibit cell specificity and are only expressed in the principal cells [14].

Sperm maturation in the epididymis is an androgen-dependent process. In the absence of androgen, sperm maturation is disrupted, and sperm do not become motile [15]. The androgen-dependency of the epididymis is suggested by the fact that several epididymal-specific genes are androgen-regulated, such as glutathione peroxidase 5 (*Gpx5*) [16], lipocalin 5 (*Lcn5*) [13] and cysteine-rich secretory protein 1 (*Crisp1*) [17]. Some reports have suggested a role for testicular factors in regulating gene expression in the epididymis. The identity of these factors has not been fully established, but some studies have reported the presence of basic fibroblast growth factor (*bFGF*) [18] and sperm-associated factors [19].

Numerous genes that are putatively involved in sperm maturation have not been fully characterized. Characterizing these genes is critical for understanding the mechanism of sperm maturation at the cellular level. Our previous gene profiling data identified epididymal genes that were affected by gonadectomy [20]. One of the interesting genes was *Spag11a* (sperm-associated antigen 11A), which requires further characterization to understand its function in the epididymis. The present study analyzed the signal peptide and other functional domains of *Spag11a* as well as its tissue specificity, expression regulation and protein localization.

## Methods

### *In silico* analyses

Members of the sperm-associated antigen cluster were identified in a microarray study of the mouse epididymis [20] and in proteomic analyses of mouse sperm [21]. We selected a member of this cluster, *Spag11a*, for further analysis. The cDNA sequence of mouse *Spag11a* was obtained from Unigene (http://www.ncbi.nlm.nih.gov/unigene/?term=Mm.229357). The Unigene database was used to determine the distribution of the ESTs in various tissues. Ensembl Mouse Gene View (http://www.ensembl.org/Mus_musculus/index.html) and UCSC Genome Bioinformatics (http://genome.ucsc.edu) were used to obtain gene structure and exon-intron boundaries. The SignalP 4.0 Server (http://www.cbs.dtu.dk/services/SignalP/) was used to predict the presence and location of signal peptide cleavage sites in the amino acid sequence [22]. InterProScan (http://www.ebi.ac.uk/Tools/pfa/iprscan/) and Motif Scan (http://myhits.isb-sib.ch/cgi-bin/motif_scan) were used to predict functional domains [23]. The Expasy Bioinformatics Resource Portal (http://web.expasy.org/compute_pi/) was used to predict protein molecular mass and isoelectric point.

### Experimental animals and RNA extraction

Adult male mice (8 weeks old) strain ddY (Deutschland, Denken and Yoken) were used in this study. All the mice were handled in accordance with the Research Ethic Committee, Faculty of Medicine, University of Indonesia. Mice were given pelleted food and water *ad libitum* in a room with controlled light (12 h of light, 12 h of darkness) and temperature (27 ± 1 C). To analyze tissue distribution, RNA was extracted from various tissues. To determine androgen dependency, 28 mice were divided into 7 groups of 4 mice each. The following groups were analyzed: control (not castrated), 6 h, 1 d, 3 d, 5 d after castration, and 3 d and 5 d after castration with testosterone replacement therapy. Castration was performed by removing both testis from the mice under anaesthesia. Testosterone replacement therapy was performed by injecting testosterone solution (Nebido, Bayer, Germany) intra muscularly at a dose of 0.5 mg/mouse/day (diluted in 0.9% NaCl) starting at the day of castration. Mice were sacrificed from each group, caput epididymides were collected and total RNA was extracted. Efferent duct ligation (EDL) was performed to analyse the influence of testicular factors. Twenty mice were divided into 5 groups of 4 mice each. The following groups were analyzed: control (un-ligated), 6 h, 1 d, 3 d and 5 d after efferent duct ligation. Ligation was carried out bilaterally by tying the efferent duct using a synthetic non-absorbable polypropylene suture 6–0 (Prolene, NJ, USA). Total caput RNA was extracted from each group. The castrations and efferent duct ligations were performed by making an incision in the scrotum under anesthesia (2.5% 2,2,2-Tribromoethanol, Avertin, Sigma, USA) diluted in 0.9% NaCl. To extract RNA, the epididymis and other tissues were snap-frozen in liquid nitrogen and stored at −80°C until RNA could be extracted. The RNA extractions were performed using the High Pure RNA Tissue Kit (Roche, Germany) according to the manufacturer’s instructions.

### Isolation of mouse spermatozoa and luminal fluid

To examine the presence of SPAG11A in the spermatozoa and luminal fluid, two mice were sacrificed and the caput epididymides, the cauda and the vas deferens were isolated and put on a watch glass containing 500 μl PBS. The caput and cauda were punctured gently using a small needle (26G x 1/2”) and incubated at 37°C for 1 hour to allow spermatozoa and luminal fluid to flow out from the duct. To isolate spermatozoa and luminal fluid from the vas deferens, while incubating in 500 μl PBS at 37°C, the ducts were gently squeezed using a round tweezers several times. After incubation, spermatozoa and the luminal fluid were separated by centrifugation at 800 × g for 5 min.

### Quantitative real-time RT-PCR

Ten nanograms of total RNA (DNase-treated) was utilized in the quantitative real-time reverse transcription PCR analysis of tissue distribution and the dependence on androgen and testicular factors. A KAPA SYBR FAST One-Step qRT-PCR Universal Kit (KAPA Biosystems, CA, USA) was used according to the manufacturer’s instructions. The primers used in this study were Spag11a_F (ACAGAGAGCGAGCCGTAAAA), Spag11a_R (AGGCACACGGTGTTTCTGAT) producing an amplicon of 113 bp. Mouse beta actin gene was used to normalize *Spag11a* expression in each sample. Primers for beta actin were beta actin_F (GATCTGGCACCACACCTTCT) and beta actin_R (GGGGTGTTGAAGGTCTCAAA) producing an amplicon of 138 bp. All primers have annealing temperatures of 60–61°C. The following program was used in the real time qRT-PCR analyses: cDNA synthesis 42°C for 10 min, reverse transcriptase inactivation 95°C for 5 min, denaturation 95°C for 15 sec, annealing 60°C for 30 sec, elongation 72°C for 60 sec. The cycle was repeated 34 times, melting ramp 50–90°C rising 0.5°C every step, acquired melting curve, and final elongation 72°C for 5 min. All samples for *Spag11a*, beta actin and standard curves were run in triplicate. Normalization values presented in each graph were obtained by dividing amplification product using *Spag11a* primers (in nanogram unit) with amplification product from beta actin (*Actb*) primers. In every qRT-PCR run, two negative controls were included, non-template negative control and minus RT negative control. Additional files show an example of detail calculation of relative gene expression (See Additional file 1: Figure S1), *Spag11a* melting curve with its RT-PCR product run on a 1% agarose gel (See Additional file 2: Figure S2) and reaction efficiency (See Additional file 3: Table S1).

### Western blot analyses

Protein samples from four different regions of the epididymis (initial segment, caput, corpus and cauda) and also from spermatozoa isolated from epididymis duct and vas deferens were extracted by solubilizing the tissue or cell in sodium dodecyl sulfate (SDS) extraction buffer [2% SDS, 10% sucrose, 0.1875 M Tris (pH 6.8)] suplemented with a protease inhibitor cocktail (Roche, Manneheim, Germany) for 5 min at 100°C. Soluble protein was obtained by centrifugation at 9000 × g for 10 min. Fifteen microgram of protein was then separated by 10% SDS-PAGE and transferred to Hybond-P PVDF membranes (Amersham, Buckinghamshire, UK). The membranes were blocked in 5% Bovine Serum Albumin in 1x TBST for 1 hour at room temperature. The membranes were then incubated overnight at 4°C with rabbit anti-human SPAG11A polyclonal antibody (Immunogen: ag9846, genebank no: BC058833) (Proteintech, USA) at a 1:1000 dilution. The antibody recognize a protein with MW = 20 kDa. The membranes were washed with 1x TBST for 3 × 5 minutes and incubated with donkey anti-rabbit IgG-conjugated horseradish peroxidase (HRP) (Santa Cruz Biotechnology, CA, USA) at a 1:5000 dilution for 1 hour at room temperature. The membranes were washed again with 1x TBST for 3 × 5 minutes, and HRP was detected using a western blot chemiluminescence detection system (Amersham, Buckinghamshire, UK). Chemiluminescence was exposed on x-ray negative film (Fuji Co, Japan).

### Immunohistochemistry

Epididymal tissue sections (5 μm thick) and sperm cells were attached to the poly-L-lysine-coated slide and used in immunohisto- and cytochemical analyses. After deparaffinization and rehydration, the sections were expose to 3% hydrogen peroxide in distilled water for 10 min. Antigen retrieval was performed by boiling the slide in 10 mM Na Citrate, pH 6,0, for 3 × 5 min and cooling it slowly to room temperature. Immunostaining was performed using the TrekAvidin-HRP Label kit (Biocare Medical, CA, USA) according to the manufacturer’s instructions. Tissues or cells were incubated overnight with a rabbit anti-human SPAG11A polyclonal antibody at a 1:200 dilution in TBS. All the incubations were performed in a humidified chamber. Color development was achieved by incubating the tissues or cells with DAB (Biocare Medical, CA, USA) and was terminated by incubating the slides in distilled water. The slides were then dehydrated in increasing concentrations of ethanol and a series of xylene solutions before being mounted with Entellan mounting medium (Merck, Darmstadt, Germany) and cover-slipped. For the immunocytochemical analyses, deparafinization, rehydration, antigen retrieval and dehydration were skipped.

### Statistical analyses

SigmaStat for Windows Version 3.10 was used to perform a one-way analysis of variance for real time qRT-PCR of the dependence on androgen and testicular factors. The difference between the means was subsequently assessed using the Holm-Sidak test. The level of significance was set at P < 0.05

## Results

### SPAG11A is a secretory protein that contains phosphorylation sites

**Figure 1:**
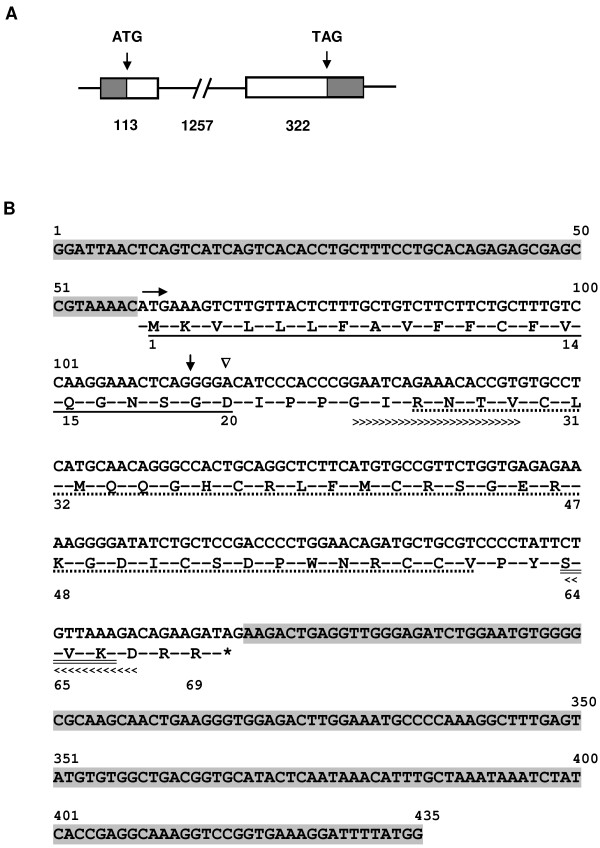
***In silico *analyses of *Spag11a *sequence. (A)** The gene structure of *Spag11a* consists of two exons (113 and 322 bp) separated by a single 1257 bp intron. The shaded areas indicate the UTR. The start codon (ATG) is located in the middle of exon 1, and the stop codon is at the end of exon 2. **(B)** The *Spag11a* transcript is 435 bp long with the start codon (ATG) located at nucleotide 59 (n59, horizontal arrow), the stop codon (TAG) at n266 (asterisk sign) and 5′ and 3′ untranslated regions (UTR, shaded area). The translation of *Spag11a* produces 69 residues with the first 20 amino acids (a1-20) constituting a signal peptide (underlined). The downward triangle indicates a cleavage site at position a20 (D: aspartate). Amino acids 26–61 (dotted underline) contain a beta defensin domain, and a24-29 (greater than signs, >>>) contain an N-myristoylation site. A protein kinase C phosphorylation site is located at a64-66 (double underlined), which overlaps with a casein kinase II phosphorylation site at a64-67 (less than signs, <<<). The boundary between exon 1 and exon 2 is indicated by a down arrow.

*Spag11a* is a 1692 bp gene located on chromosome 8 that consists of 2 exons and 1 intron. Exon 1 is 113 bp long, and exon 2 is 322 bp long. The exons are separated by a 1257 bp intron (Figure 1A). *Spag11a* encodes a protein of 69 amino acids (Figure 1B) with predicted molecular mass of 7.9 kDa and isoelectric point of 9.03. SPAG11A belongs to the beta defensin family and has a putative function in the defense response to bacteria. The beta defensin domain is located at amino acids (aa) 26–61. To determine whether SPAG11A is a secretory protein, as is typical for proteins involved in sperm maturation, we looked for a signal peptide sequence using SignalP program. The results indicated that SPAG11A contained sequence of the signal peptide in the first twenty amino acids of the N-terminus of the polypeptide (Figure 1B). The cleavage site was located at twentieth amino acid, an aspartic acid (D). This indicated that SPAG11A is a secretory protein that may be secreted into the epididymal lumen and may interact with spermatozoa. The details of the signal peptide analysis can be found in an additional file (See Additional file 4: Figure S3). We also noticed that SPAG11A contained an N-myristoylation site at amino acids 24–29 and two potential phosphorylation sites for protein kinase C (aa 64–66) and casein kinase II (aa 64–67) (Figure 1B). The result of the motif scan analysis is available in an additional file (See Additional file 5: Figure S4).

### Spag11a is expressed exclusively in the caput region of the mouse epididymis

**Figure 2:**
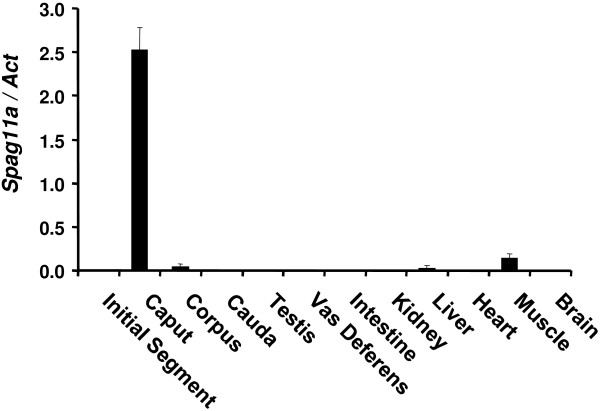
**Quantitative real-time RT-PCR analyses for *Spag11a *tissue distribution.** Total RNA from various tissues, indicated on the x-axis, was utilized to measure the relative expression level of *Spag11a*. The results revealed that *Spag11a* was expressed exclusively in the caput epididymis, although a low level of expression was detected in the corpus epididymis, liver and muscle. The expression in the other examined tissues was close to background and was nearly undetectable. The error bars indicate the SEM (n = 3).

Previous data have demonstrated that epididymal genes involved in sperm maturation process tend to be expressed specifically in the epididymis. We analyzed the tissue distribution of *Spag11a* by isolating total RNA from various tissues, including the four regions of the epididymis: the initial segment, the caput, the corpus and the cauda. The RNA was analyzed by quantitative real-time RT-PCR. In each tissue, *Spag11a* expression was normalized to the expression of the mouse housekeeping gene, beta-actin. The results established that *Spag11a* was exclusively expressed in the epididymis. Very low expression was detected in muscle and liver, whereas nearly undetectable background expression was observed in the testis, vas deferens, intestine, kidney, heart and brain (Figure 2). Interestingly, *Spag11a* exhibited a region-specific expression pattern, it was only expressed in the caput region. Very low expression was detected in the corpus and the cauda in which the expression was only 0.8% and 0.4%, respectively, of the level in the caput. These results suggested that *Spag11a* may have a specific role in creating a regional environment in the caput that is suitable for sperm maturation. The relative expression calculation is available in an additional file (See Additional file 6: Table S2).

### Spag11a is regulated by androgen and testicular factors

**Figure 3:**
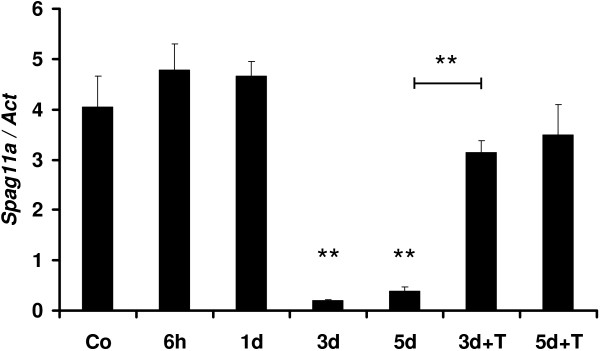
**Quantitative real-time RT-PCR analyses of the androgen dependence of *Spag11a *in the epididymis.** Mice were castrated, and the epididymides were isolated for RNA extraction after 6 h, 1 d, 3 d, 5 d, and also 3 d, 5 d castration with testosterone replacement therapy (3d + T, 5d + T). The expression levels were normalized to beta-actin. The error bars represent the standard error of the mean (SEM, n = 3). The results demonstrated that *Spag11a* was modestly up-regulated at 6 hours to 1 day following castration before being significantly down-regulated (P < 0.001) 3–5 days after castration. The expression levels after testosterone (T) injection (0.5 mg/mouse/day) for 3d + T and 5d + T were similar to control non castrated (Co). The difference between the expression levels at 3–5 d and 3d + T was significant (P < 0.001), confirming the effectiveness of the T replacement therapy.

Because sperm maturation in the epididymis is androgen-dependent, we tested *Spag11a* for androgen dependency by performing a castration/gonadectomy experiment. The results indicated that *Spag11a* was slightly up-regulated 6 hours after gonadectomy but was not significantly different from the control. The expression was maintained for 1 day after gonadectomy before being dramatically down-regulated on days 3 and 5. The lowest level was achieved on day 3, when the expression was 19-fold lower than in the control group (P < 0.01). Interestingly, exogenous testosterone maintained a nearly normal expression level through days 3 and 5 (Figure 3). The effectiveness of T-replacement therapy in our experiment was confirmed by testing a known androgen-dependent gene *Defb42*[24] which is presented in an additional file (See Additional file 7: Figure S5). This suggested that *Spag11a* is primarily regulated by circulating androgen. The average relative expression levels for each group are available in an additional file (See Additional file 8: Table S3).

**Figure 4:**
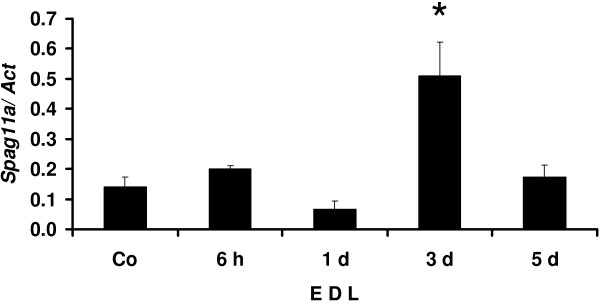
**Quantitative real-time RT-PCR analyses for efferent duct ligation (EDL) to determine the involvement of testicular factors in regulating *Spag11a*.** The efferent ducts connecting the testis and the epididymis were ligated to prevent testicular fluid from entering the epididymis. Total caput epididymal RNA from both sides were isolated at 6 h, 1 d, 3 d and 5 d after the ligation. Unligated epididymis was used as a control (Co). Total RNA from each group was analyzed by quantitative real-time RT-PCR. The expression level in each group was normalized to beta-actin. The results showed that *Spag11a* expression was not affected at 6 h to 1 d. after the ligation. *Spag11a* was transiently up-regulated at 3 d ligation (P < 0.01) before returning to the normal level at day 5. The error bars represent the SEM (n = 3).

Besides androgen, testicular factors also regulate gene expression in the epididymis [18,19]. To confirm whether luminal factors were involved, we performed efferent duct ligation (EDL) by tying the efferent ducts that connect the testis with the proximal part of the epididymis. Our data showed that the lack of testicular factors did not affect *Spag11a* expression at 6 hours to 1 day after EDL. Interestingly, we observed 3.6 folds transient up-regulation of *Spag11a* compared to the control levels at 3 days after EDL (P < 0.05) before it decreased again almost to the normal levels at 5 days after EDL (Figure 4). The relative expression levels for each group are available in an additional file (See Additional file 9: Table S4).

### SPAG11A protein is expressed in the caput region of the epididymis

**Figure 5:**
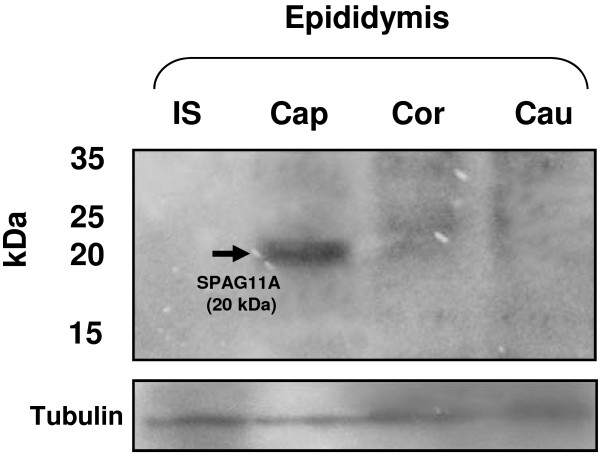
**Western blots analyses confirmed the presence of SPAG11A protein in the epididymis.** Fifteen micrograms of protein from each region of the epididymis was separated by 10% SDS-PAGE and transferred to PVDF membranes. SPAG11A was detected with a rabbit anti-human SPAG11A antibody. IS = initial segment; Cap = Caput; Cor = Corpus; Cau = Cauda. The results showed the presence of SPAG11A (MW = 20 kDa, indicated by an arrow) in the mouse caput epididymis. A very weak and smear band of SPAG11A was detected in the corpus, whereas no signals were observed in the initial segment and cauda. The same membrane was stripped and re-probed with antibody against α-tubulin as a loading control.

To confirm that *Spag11a* encodes a protein that is present in epididymal tissue, we performed western immunoblotting using protein samples from each epididymal region (initial segment, caput, corpus and cauda). The result showed SPAG11A was present in the caput region of the epididymis but not in other regions (Figure 5). SPAG11A was detected as a single band with molecular weight of 20 kDa. A very weak and smear band of SPAG11A was detected in the corpus region. This result corresponded with our quantitative real-time RT-PCR on tissue distribution analysis in which the highest expression of *Spag11a* was observed in the caput region and very low expression was detected in the corpus (Figure 2).

### SPAG11A is expressed specifically in the principal cells of the caput epididymis

**Figure 6:**
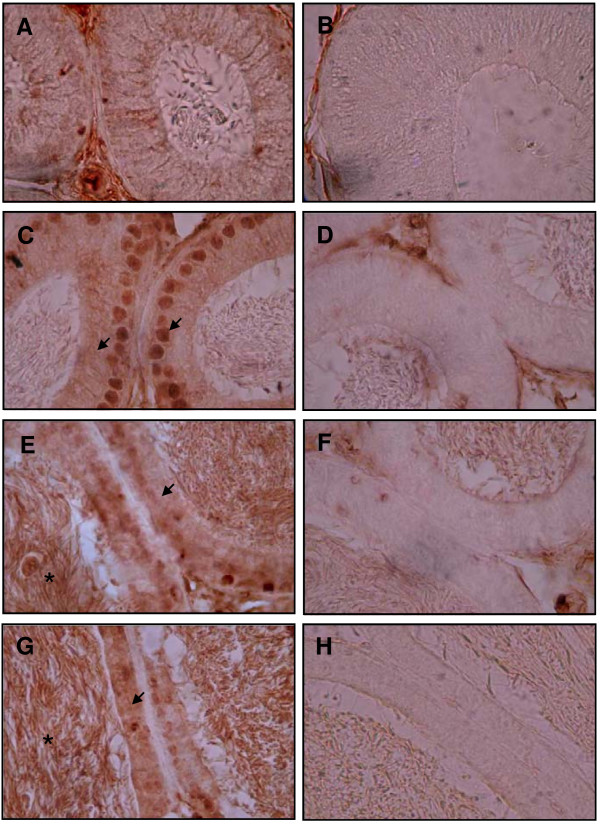
**Immunohistochemistry analyses of SPAG11A in the four regions of the mouse epididymis.** The epididymis was isolated, fixed in 4% paraformaldehyde and cut transversely in 5 μm-thick sections. An antibody against human SPAG11A was used to examine the expression of SPAG11A protein in the four epididymal regions. **(A)** SPAG11A was not present in the initial segment with no staining was detected in the nucleus and cytoplasm of the epithelial cells. **(C)** SPAG11A was detected both in the nucleus and cytoplasm of the principal cells of the caput. **(E** and **G)** Cytoplasmic staining was also detected in the corpus and cauda, respectively. Positive staining was indicated by arrows. **(B**, **D**, **F**, **H)** Specificity of staining in each region was assessed by comparing with negative controls for initial segment, caput, corpus and cauda, respectively. The negative controls were treated in the same manner as the other samples except PBS was used instead of the primary antibody against SPAG11A. It is interesting to note that positive staining was also detected in the sperm cells inside the lumen of corpus and cauda (*). The images were observed at 1000× magnification.

**Figure 7:**
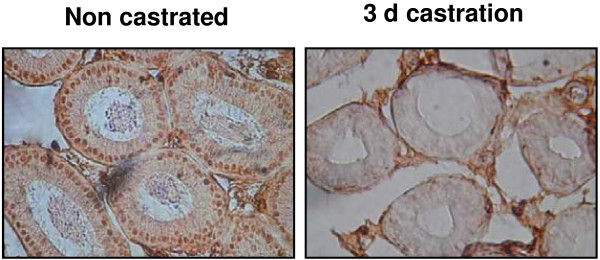
**Immunohistochemistry to confirm the dependence of SPAG11A protein expression on androgen.** Staining using SPAG11A polyclonal antibody was performed on caput epididymal tissue sections from a normal non castrated mouse and 3 d castration. The results showed consistent staining in the nucleus and faint staining in the cytoplasm of the caput principal cells in the non castrated mice and the staining disappeared after 3 d castration. The images were observed at 400× magnification.

There are several types of epithelial cells in the epididymal duct, each with a specific function. Proteins that are involved in epididymal sperm maturation are typically expressed in a cell type-specific manner. We performed immunohistochemistry to localize SPAG11A expression in the epididymal duct using a rabbit anti-human SPAG11A antibody. Interestingly, positive staining was detected in the nucleus and also in the cytoplasm of the principal cells residing in the caput region of the mouse epididymis (Figure 6C), whereas cytoplasmic staining was detected in the corpus and cauda regions (Figure 6E and 6G). The staining was absent in the initial segment (Figure 6A) indicating region-specific expression of the SPAG11A. Moreover, we also observed SPAG11A staining in the sperm in the lumen of corpus and cauda, but not in the lumen of initial segment and caput. This result suggested that SPAG11A start to be secreted in the caput region and bind to the sperm in the corpus and cauda. The specificity of the staining was confirmed by including a negative control for initial segment, caput, corpus and cauda (Figure 6B, D, F, H, respectively). In addition, we also confirmed the regulation of SPAG11A protein expression by androgen using immunohistochemistry which demonstrated the lost of staining in the principal cells of the caput epididymis after 3 d castration (Figure 7).

### SPAG11A protein is present in the spermatozoa and epididymal fluid

**Figure 8:**
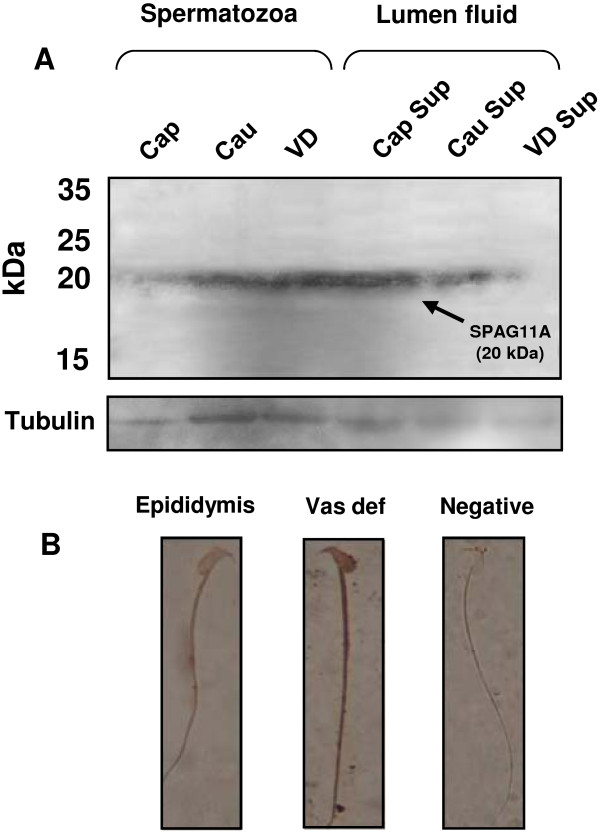
**Western blots and immunocytochemistry revealed the presence of SPAG11A in mouse spermatozoa and epididymal luminal fluid. (A)** Protein was extracted from mouse spermatozoa isolated from the caput epididymis (Cap), cauda (Cau) and vas deferens (VD) and luminal fluid from the caput (Caput supernatant, Cap Sup), cauda fluid (Cau Sup) and vas deferens fluid (VD Sup). Fifteen micrograms of protein was separated by 10% SDS-PAGE and transferred to PVDF membranes. The target protein was detected with a rabbit polyclonal anti-human SPAG11A antibody. The results revealed that SPAG11A (20 kDa, indicated by an arrow) was detected weakly in sperm isolated from the caput region, but more SPAG11A was present in cauda and vas deferens sperm. In the luminal fluid, SPAG11A was detected strongly in the caput, reduced towards the cauda and no signal was detected in the vas deferens. The membrane was subsequently stripped and re-probed with an antibody against mouse alpha tubulin as a loading control. **(B)** Immunocytochemistry on the mouse sperm isolated from the epididymis and vas deferens using the same primary antibody. The results indicated more intense staining in the vas deferens sperm compared to the epididymal sperm. Sperm incubated only with a secondary antibody was used as a negative control. The images were observed at 1000x magnification.

The process of sperm maturation in the epididymis occurs by interaction between spermatozoa and proteins secreted by epididymal epithelium. To examine whether SPAG11A is actually secreted in the epididymal fluid and bind to the spermatozoa, we checked the presence of the protein in spermatozoa taken from two regions of the epididymis (caput and cauda regions) and vas deferens. Western immunobloting results indicated that SPAG11A was detected weakly in the caput epididymal spermatozoa and the amount of protein increased in the cauda and vas deferens (Figure 8A, left panel). Interestingly, SPAG11A was also detected in the lumen fluid of the caput and the cauda but not in the vas deferens (Figure 8A, right panel). We also performed immunocytochemistry to examine if the protein bind to the spermatozoa upon exit from the epididymis. The result showed that there was a significant increase in staining intensity in the spermatozoa taken from the vas deferens compared to the epididymal spermatozoa indicating more SPAG11A deposited in the spermatozoa when they leave the epididymis (Figure 8B).

## Discussion

Spermatozoa are transcriptionally and translationally silent cells. The development from immature to mature cells capable of fertilizing an oocyte depends on post-translational modification of pre-existing proteins. These modifications occur via interactions with proteins secreted by the epididymal epithelium while sperm traverse the epididymis. We characterized *Spag11*a in the mouse epididymis at both the RNA and protein levels to obtain data on the putative role of this gene in sperm maturation.

SPAG11A is a member of the beta defensin family and contains a signal peptide sequence. This protein family has antimicrobial activity and is involved in host defense [25]. The defensin gene family has evolved by repeated gene duplication and divergence, including functional diversification [26]. Reproductive functions are suggested by the surface localization of several defensins, including SPAG11 [27], DEFB118 [28] and DEFB126 [29], on sperm. SPAG11E, also known as *Bin1b*, is known to promote motility in immature spermatozoa in the caput epididymis via a calcium uptake-dependent mechanism [30]. Alternative spliced transcript have produced multiple *Spag11* isoforms in epididymal epithelial cells. Although many members of the beta defensin family have been characterized, the exact role of *Spag11a* in the mouse epididymis is unknown. The presence of a signal peptide sequence suggested that mouse SPAG11A is a secretory protein that may be involved in sperm maturation. Moreover, we discovered that SPAG11A contains phosphorylation sites such that upon binding to sperm, the cell can be modified by protein kinases. This corresponded with a finding that during transit through the epididymis, spermatozoa undergo changes in tyrosine phosphorylation [31]. Concerning binding to sperm, we identified a myristoylation site in SPAG11A. The presence of this potential N-myristoylation site suggested that the protein may covalently bind to the plasma membrane of sperm [32,33].

Our data demonstrated that mouse *Spag11a* was expressed exclusively in the epididymis, not in other tissues such as the testis, vas deferens, intestine, kidney, liver, heart, muscle and brain. Moreover, mouse *Spag11a* exhibited a region-specific expression pattern and was mainly present in the caput region. This is similar to several genes that are important for sperm maturation such as *Rnase10*[34,35], *Crisp4*[36,37], and *Crisp1*[17], which are exclusively expressed in the initial segment, caput and corpus/cauda, respectively. This region-specific expression is important to create specific environments for sperm maturation. Although the highest expression was detected in the caput, low expression levels were detected in corpus epididymis, muscle and liver (Figure 2). This is possible because the beta defensin family has diverse members, and some of which function in the muscle [38]. A study by Yamaguchi *et al.* also showed multiple epididymis-specific beta defensin isoforms in human and mice, including mouse EP2e, which is a synonym of *Spag11a*, with expression in caput, corpus and cauda [39]. Our study is first to show *Spag11a* expression specificity in the caput region both at the transcript and protein levels.

Sperm maturation in the epididymis is androgen-dependent. Our data demonstrated that *Spag11a* was slightly up-regulated 6 hours to 1 day after castration/gonadectomy before being dramatically down-regulated 3 days after castration. The androgen dependence was confirmed with a rescue experiment in which exogenous testosterone was injected daily into castrated mice. The results indicated that exogenous testosterone could maintain *Spag11a* expression at nearly normal levels on days 3 and 5 after castration. This indicated that *Spag11a* was primarily regulated by circulating androgen. Moreover, our study in the regulation of *Spag11a* by androgen was also confirmed by immunohistochemistry which demonstrated a total lost of staining in the caput principal cells after 3 d castration. This is similar to the regulation of several genes involved in sperm maturation, such as *Eppin* (epididymal protease inhibitor) [40], *Pate* (cysteine rich prostate and testis expressed protein) [41] and the serine protease inhibitor *HongrES1*[42]. EPPIN is an antimicrobial cysteine-rich protein that contains both Kunitz and whey acidic protein (WAP) domains and is a target for male contraception because of its critical role in sperm motility [43].

In addition to androgen, epididymal genes are also regulated by testicular factors. Because the observed recovery of *Spag11a* expression after gonadectomy/castration with testosterone replacement was slightly lower than that in the control non-gonadectomy group, we tested the possibility that testicular factors were involved in *Spag11a* regulation. By using efferent duct ligation (EDL), we blocked testicular fluid (lumicrine factor) from entering the epididymis while preserving testosterone supply from both testis. The result from the EDL experiment showed that the lack of testicular fluid did not affect *Spag11a* expression at 6 hours to 1 day after the ligation. Interestingly, *Spag11a* was transiently up-regulated at 3 days after EDL before down-regulated back to the level of control at 5 days after EDL (Figure 4). The reason for the transient increase is not known, but we hypothesize that it may be caused by initiation or the onset of apoptosis within the cell which somehow stimulates a temporary up-regulation of *Spag11a* in response to the process. This notion is based on previous studies that orchidectomy and efferent duct ligation induces apoptotic cell death in the caput epididymis that reach maximum at day 3 [44,45]. Several epididymal genes are regulated by testicular factors, including gamma-glutamyltransferase 1 (*Ggt1*, regulated by fibroblast growth factor (FGF)) [46], 5-alpha reductase (regulated by androgen binding protein (ABP)) [47] and proenkephalin (*Penk*, regulated by sperm-associated factors) [19]. The primary regulation of caput-specific *Spag11a* by androgen confirmed our previous report that epididymal genes enriched in the initial segment are more dependent on testicular factors whereas androgen regulates most of the caput-enriched genes [20].

We also analyzed whether the expression of *Spag11a* mRNA was consistent with the protein expression. We performed western immunoblotting using protein extracts from four different regions of the mouse epididymis. SPAG11A protein was detected with a rabbit anti-human SPAG11A polyclonal antibody. The results demonstrated that SPAG11A was only present in the caput region (Figure 5) which confirmed the antibody specificity. Because the protein was present in the caput epididymis, we performed immunohistochemistry to localize SPAG11A at the subcellular level. SPAG11A was localized in the nucleus and cytoplasm of the principal cells in the caput region, whereas only cytoplasmic staining was detected in the corpus and cauda. This is in agreement with the tissue distribution analyses using qRT-PCR in which the highest expression was in caput whereas very weak expression was detected in the corpus and cauda (Figure 2). The cell-specific expression of SPAG11A confirmed its putative role as a secretory protein that creates a microenvironment suitable for sperm maturation. Principal cells contain secretory apparatuses, such as endoplasmic reticulum, Golgi and secretory granules, and endocytic apparatuses, including coated pits, endosomes, multivesicular bodies and lysosomes. Therefore, the primary functions of principal cells are to synthesize and secrete proteins and to perform endocytosis [4]. This is particularly interesting because a signal peptide sequence was identified in the first twenty amino acids of the N-terminus of SPAG11A (Figure 1), which is characteristic of secretory proteins. An epididymal gene known to have a cell type-specific expression pattern is cystic fibrosis transmembrane conductance regulator (CFTR) which is also expressed specifically in the principal cell to release ATP into epididymal lumen [48].

It is intriguing to observe that SPAG11A, with a signal peptide indicating a secretory protein was localized in the nucleus. Although this is an unusual phenomenon, examples of similar protein behaviour do exist. A study of ADAMTS13, a secreted zinc metalloprotease involved in an array of processes including development and angiogenesis, detected the protein in the nucleus of liver cells [49]. Other metalloproteinases, MMP-2 [50] and MMP3 [51], which are involved in extracellular matrix remodeling, were detected in the nucleus of cardiac myocytes and chondrocytic cells, respectively. This suggests intracellular role for these secreted proteins. MMP3, for instance, behaves as a proteinase that degrades matrix components following its secretion, while behaving as a transcription factor when present within the nucleus. It is also possible that SPAG11A has another intracellular role and it shuttles between nucleus and cytoplasm through nuclear pore complex. This is particularly interesting if we correlate it with transient up-regulation of *Spag11a* at 3 d following efferent duct ligation (EDL) and returned to the normal level at 5 d post EDL. That coincided with a major change in the caput epididymal cell at day 3 following orchidectomy or EDL, particularly the onset of apoptosis [44,45].

Our study is the first to show that SPAG11A is a secretory protein that is present in the epididymal fluid and spermatozoa taken from the cauda epididymis and vas deferens. SPAG11A is secreted mainly by principal cells of the caput epididymis and the protein was detected in epididymal fluid but minimum amount of protein was detected in the vas deferens fluid. The reduced amount of protein in the vas deferens luminal fluid indicating that most of the protein may have bound to the sperm cell. Secreted from the caput and to some extent from corpus and cauda region, the protein subsequently bind to the spermatozoa. An increasing amount of SPAG11A was detected in the protein extracted from cauda and vas deferens sperm compared to the caput sperm (Figure 8A). Furthermore, by using immunocytochemistry, we also showed more intense SPAG11A staining in the sperm cells taken from vas deferens compared to the epididymal sperm, confirming more SPAG11A protein deposited to the sperm upon exit from the epididymis. We believe that data from this study is important for a further study to determine the role of SPAG11A during epididymal sperm maturation and fertilization.

## Conclusions

We have characterized *Spag11a* in the mouse epididymis to determine its potential role in sperm maturation. The presence of a signal peptide and several domains for protein modification, such as kinase binding sites, indicated that SPAG11A is a secretory protein that enables post-translational modifications of sperm. The tissue- and region-specific expression in the caput epididymis and regulation by androgen suggested that *Spag11a* may be involved in creating a microenvironment suitable for sperm maturation. Furthermore, the presence of SPAG11A protein mainly in the principal cells of the caput, epididymal luminal fluid and spermatozoa taken from the cauda and vas deferens corroborate the idea that SPAG11A protein is secreted into the lumen of the epididymis and binds to the spermatozoa. Additional studies are required to verify the exact role of *Spag11a* in the sperm maturation process.

## Abbreviations

DAB: Diaminobenzidine; EST: Expressed sequence tag; HRP: Horseradish peroxidase; PBS: Phosphate buffered saline; PVDF: Polyvinylidene difluoride; SDS-PAGE: Sodium dodecyl sulfate-polyacrylamide gel electrophoresis; TBS: Tris-buffered saline; TBST: TBS-Tween; ABP: Androgen binding protein; ADAMTS13: ADAM metallopeptidase with thrombospondin type 1 motif 13; bFGF: Basic fibroblast growth factor; CFTR: Cystic fibrosis transmembrane conductance regulator; Crisp1: Cysteine-rich secretory protein 1; Eppin: Epididymal protease inhibitor; Ggt1: Gamma-glutamyltransferase 1; Gpx5: Glutathione peroxidase 5; Lcn5: Lipocalin 5; MMP-2: Matrix metalloproteinase-2; MMP3: Matrix metalloproteinase 3; Pate: Prostate and testis expressed protein; Penk: Proenkephalin; Spag11a: Sperm associated antigen 11a; WAP: Whey acidic protein.

## Competing interests

The authors declare that they have no competing interests.

## Authors’ contributions

DAP contributed in designing and performing the gene expression analyses, androgen dependency experiments and immunocytochemistry and by drafting the manuscript. EL contributed to the gene expression analyses, androgen dependency experiments, protein analyses and immunocytochemistry. PS and YHM participated in designing the experiments and interpreting the data. PSH contributed to designing the experiments, interpreting the data and revising the manuscript. All authors read and approved the final manuscript.

## Authors’ information

DAP is a reproductive biologist. He obtained his PhD from The University of Turku, Finland, where he studied epididymal-specific genes that are regulated by androgen and lumicrine factors. He received postdoctoral training in the Reproductive Science Group at The University of Newcastle, NSW, Australia, studying prolactin as a prosurvival factor for human sperm. He has published 7 papers in his primary field of research: the epididymis and human sperm project. DAP is currently a researcher and lecturer at the Faculty of Medicine, University of Indonesia, Jakarta, Indonesia. PSH is a Professor of Molecular Biology who obtained his PhD from the University of Queensland, Australia. PSH is currently a researcher and lecturer at the Faculty of Medicine, University of Indonesia, Jakarta, Indonesia.

## Supplementary Material

Additional file 1: Figure S1 Example of detail calculation of relative gene expression for *Spag11a* androgen dependence analyses. Standard graphs were generated for mouse *Spag11a* and *Actb* house keeping gene by making serial dilution of RNA samples from caput epididymis (1000, 100, 10, 1, 0.1, 0.01 ng). Slope and intercept from the standard curve were used to calculate log ng from average CT value. The ratio between log ng from *Spag11a* and *Actb* was used to represent relative expression of *Spag11a*. Click here for file

Additional file 2: Figure S2 Melting curve from qRT-PCR using *Spag11a* primers and RT-PCR product run on a 1% agarose gel. **(A)** Real-time qRT-PCR using *Spag11a* primers produced a single-peak melting curve indicating specificity of the primer **(B)** The specificity of the primers was further confirmed by running the product on a 1% agarose gel. *Spag11a* primers produced a single band at 113 bp and the best annealing temperature was 60°C. RT-PCR product using beta actin (*Actb*) primers was included as a control. *Actb* primers produced a single band at 138 bp and the annealing temperature was 60°C. Click here for file

Additional file 3: Table S1 Reaction efficiency of real-time qRT-PCR using *Spag11a* and *Actb* primers. Both primers used in this study produced reaction efficiency of 0.92 for *Spag11a* and 0.90 for *Actb* (highlighted with yellow color). Click here for file

Additional file 4: Figure S3 Analyses of SPAG11A signal peptide using SignalP program. The green line indicates the S score (signal peptide). The peak of the red vertical line (C score) indicates a predicted cleavage site. The blue line (Y score) constitutes a combination of the S and C scores for a better prediction of a cleavage site. A cleavage site is located where the green line meets the highest peak (highest score) of the red line. In SPAG11A, the cleavage site is located at amino acid 20 (D: aspartate). Click here for file

Additional file 5: Figure S4 Result of the motif scan analyses of SPAG11A. Several domains were predicted using motif scan analyses available at http://myhits.isb-sib.ch/cgi-bin/motif_scan, including casein kinase II phosphorylation sites at position 64–67, an N-myristoylation site at 24–29, a protein kinase C phosphorylation site at 64–66 and a beta defensin domain at 26–61. Click here for file

Additional file 6: Table S2 Relative expression of *Spag11a* in various tissues. The expression level of *Spag11a* was normalized to that of beta-actin in a triplicate experiment (I, II and III). The averages of the ratios and the standard error of the means (SEM) were used to draw a graph presented in Figure 2. Click here for file

Additional file 7: Figure S5 Real-time qRT-PCR analyses to check androgen dependence for *Defb42*. **(A)** Other known androgen dependent gene, *Defb42* was used as a positive control for the gonadectomy (castration) and testosterone replacement therapy experiment. The same RNA samples for androgen dependence analyses in *Spag11a* were utilized to check *Defb42* in the control (un-castrated), 6 h, 1 d, 3 d, 5 d, 3d + T and 5d + T. *Defb42* mRNA was dramatically down-regulated at 6 h after castration and reach the lowest level at 3 d. T-replacement therapy was able to maintain the expression level close to normal level at 3d + T and 5d + T suggesting a reliable experiment set up for analyzing androgen regulation of *Spag11a.***(B)** Detail calculation of *Defb42* relative expression after castration and T-replacement therapy. Click here for file

Additional file 8: Table S3 Relative expression of *Spag11a* in the androgen dependence experiment. The expression of *Spag11a* in control (uncastrated, Co), 6 h, 1 d, 3 d, 5 d after gonadectomy or castration and 3 d and 5 d castration with testosterone replacement therapy were normalized with beta actin. The average of the ratio of *Spag11a* to beta actin and the standard error of the mean (SEM) were used to draw a graph presented in Figure 3. Click here for file

Additional file 9: Table S4 Relative expression of *Spag11a* in the efferent duct ligation (EDL) experiment. The expression levels of *Spag11a* in the control group (unligated) and 6 h, 1 d, 3 d, and 5 d following efferent duct ligation were normalized to beta actin. The average ratio from a triplicate experiment (I, II and II) and the standard error of the mean (SEM) were used to draw a graph presented in Figure 4. Click here for file
