# Supplementary figures and images for: Sperm-associated antigen 11A is expressed exclusively in the principal cells of the mouse caput epididymis in an androgen-dependent manner
Source: Reprod Biol Endocrinol. 2013 Jul 1;11:59. doi: 10.1186/1477-7827-11-59 (PMC3710511; doi:10.1186/1477-7827-11-59)

## Slide 1
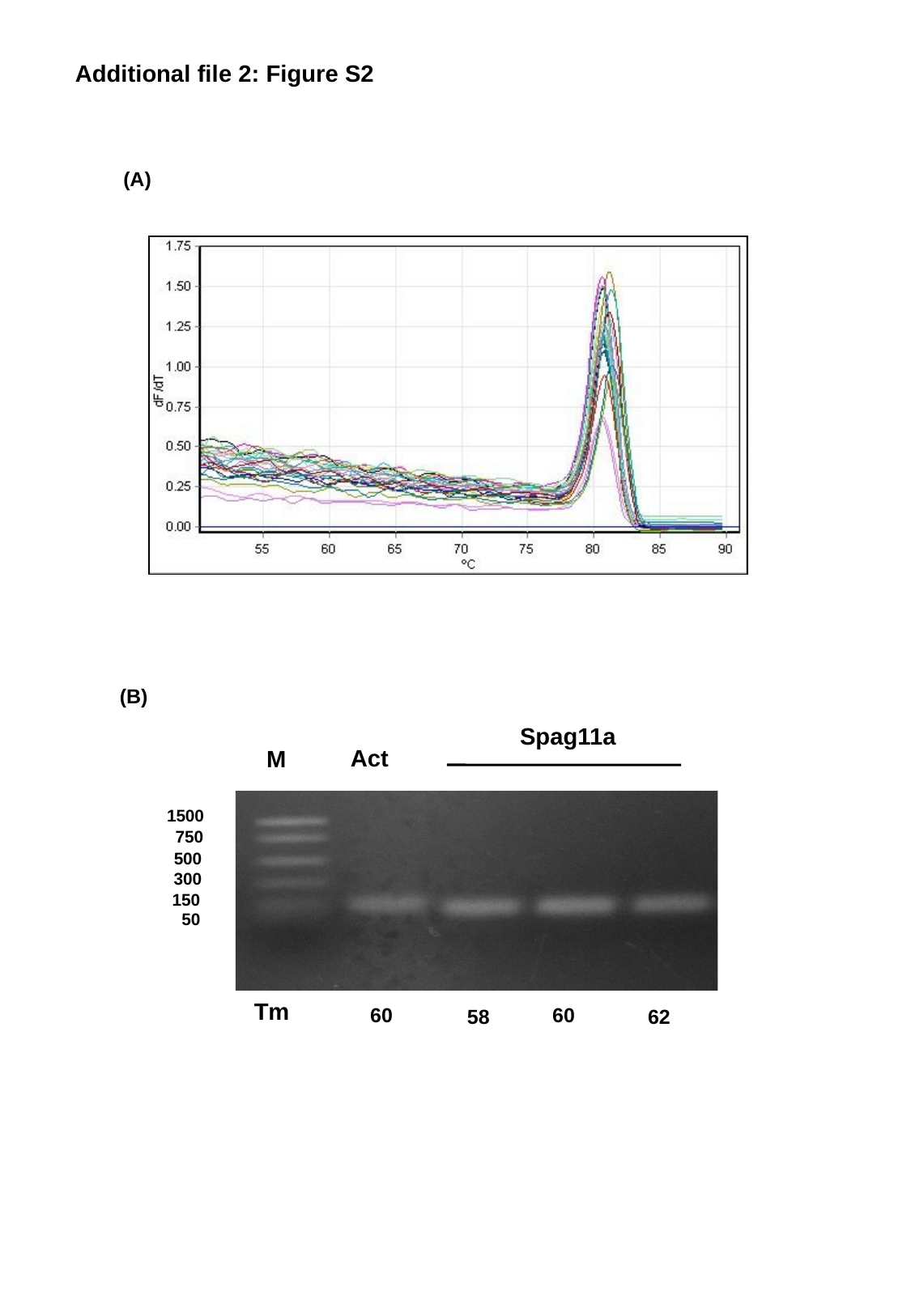

Additional file 2: Figure S2
(A)
(B)
Spag11a
Act
M
1500
750
500
300
150
50
Tm
60
60
58
62

Supplement: Additional file 2: Figure S2 — Melting curve from qRT-PCR using Spag11a primers and RT-PCR product run on a 1% agarose gel. (A) Real-time qRT-PCR using Spag11a primers produced a single-peak melting curve indicating specificity of the primer (B) The specificity of the primers was further confirmed by running the product on a 1% agarose gel. Spag11a primers produced a single band at 113 bp and the best annealing temperature was 60°C. RT-PCR product using beta actin (Actb) primers was included as a control. Actb primers produced a single band at 138 bp and the annealing temperature was 60°C. [file 1477-7827-11-59-S2.ppt]

## Slide 1
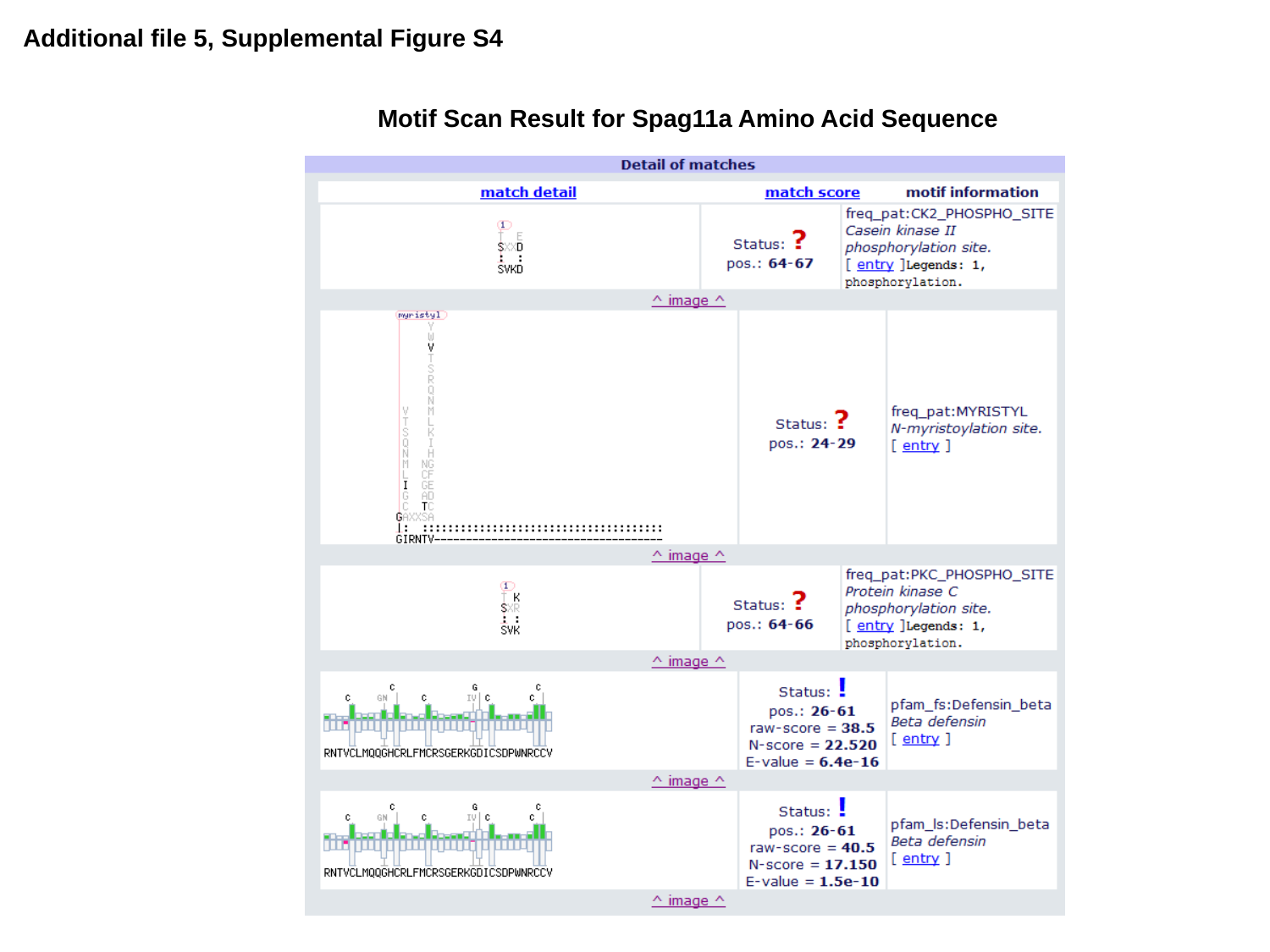

Additional file 5, Supplemental Figure S4
Motif Scan Result for Spag11a Amino Acid Sequence

Supplement: Additional file 5: Figure S4 — Result of the motif scan analyses of SPAG11A. Several domains were predicted using motif scan analyses available at http://myhits.isb-sib.ch/cgi-bin/motif_scan, including casein kinase II phosphorylation sites at position 64–67, an N-myristoylation site at 24–29, a protein kinase C phosphorylation site at 64–66 and a beta defensin domain at 26–61. [file 1477-7827-11-59-S5.ppt]

## Slide 1
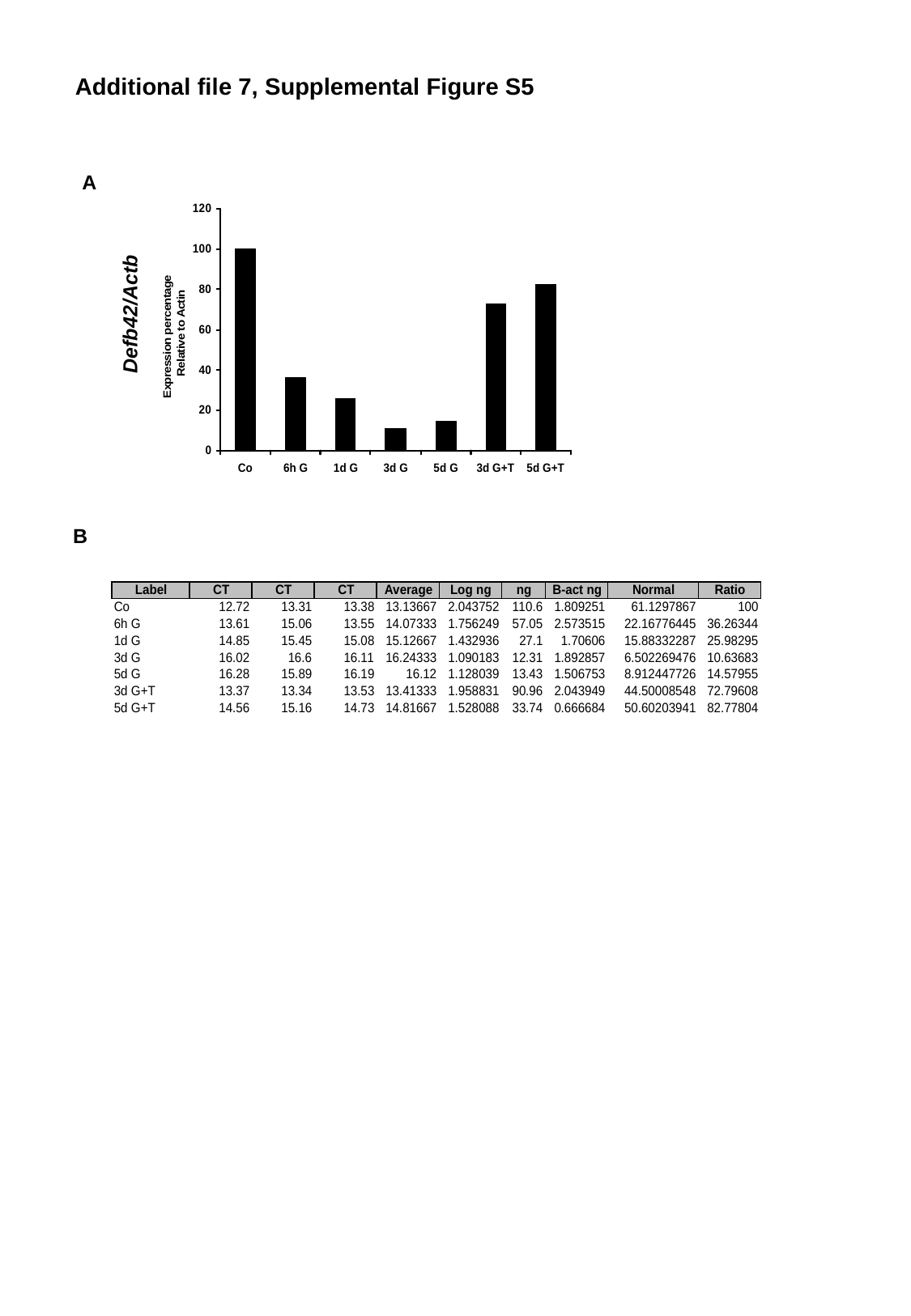

Additional file 7, Supplemental Figure S5
A
Defb42/Actb
B

Supplement: Additional file 7: Figure S5 — Real-time qRT-PCR analyses to check androgen dependence for Defb42. (A) Other known androgen dependent gene, Defb42 was used as a positive control for the gonadectomy (castration) and testosterone replacement therapy experiment. The same RNA samples for androgen dependence analyses in Spag11a were utilized to check Defb42 in the control (un-castrated), 6 h, 1 d, 3 d, 5 d, 3d + T and 5d + T. Defb42 mRNA was dramatically down-regulated at 6 h after castration and reach the lowest level at 3 d. T-replacement therapy was able to maintain the expression level close to normal level at 3d + T and 5d + T suggesting a reliable experiment set up for analyzing androgen regulation of Spag11a.(B) Detail calculation of Defb42 relative expression after castration and T-replacement therapy. [file 1477-7827-11-59-S7.ppt]
