# Supplementary material for: Sperm-associated antigen 11A is expressed exclusively in the principal cells of the mouse caput epididymis in an androgen-dependent manner
Source: Reprod Biol Endocrinol. 2013 Jul 1;11:59. doi: 10.1186/1477-7827-11-59 (PMC3710511; doi:10.1186/1477-7827-11-59)
Supplement: Additional file 3: Table S1 — Reaction efficiency of real-time qRT-PCR using Spag11a and Actb primers. Both primers used in this study produced reaction efficiency of 0.92 for Spag11a and 0.90 for Actb (highlighted with yellow color). [file 1477-7827-11-59-S3.doc]

#### Additional file 3: Table S1

#### Reaction Efficiency for Spag11a

#### Experiment Information

| Run Name | Spag11a gonadektomi |
| --- | --- |
| Run Start | 4/3/2012 12:05:45 PM |
| Run Finish | 4/3/2012 2:21:06 PM |
| Operator | Evelyn |
| Notes |  |
| Run On Software Version | Rotor-Gene 2.0.2.4 |
| Run Signature | The Run Signature is valid. |
| Gain Green | 5. |

#### Quantitation Information

| Threshold | 0.0297 |
| --- | --- |
| Left Threshold | 1.000 |
| Standard Curve Imported | No |
| Standard Curve (1) | conc= 10^(-0.285*CT + 5.197) |
| Standard Curve (2) | CT = -3.509*log(conc) + 18.234 |
| Reaction efficiency (*) | 0.92756 (* = 10^(-1/m) - 1) |
| M | -3.50867 |
| B | 18.2345 |
| R Value | 0.99835 |
| R^2 Value | 0.9967 |
| Start normalising from cycle | 1 |
| Noise Slope Correction | No |
| No Template Control Threshold | 0% |
| Reaction Efficiency Threshold | Disabled |
| Normalisation Method | Dynamic Tube Normalisation |
| Digital Filter | Light |
| Sample Page | Page 1 |
| Imported Analysis Settings |  |

#### Profile

| Cycle | Cycle Point |
| --- | --- |
| Hold @ 42°c, 10 min 0 secs |  |
| Hold 2 @ 95°c, 5 min 0 secs |  |
| Cycling (34 repeats) | Step 1 @ 95°c, hold 15 secs |
|  | Step 2 @ 60°c, hold 30 secs |
|  | Step 3 @ 72°c, hold 60 secs, acquiring to Cycling A([Green][1][1]) |
| Melt (50-90°c) , hold secs on the 1st step, hold 10 secs on next steps, Melt A([Green][1][1]) |  |
| Hold 3 @ 72°c, 5 min 0 secs |  |
| Hold 4 @ 25°c, 1 min 0 secs |  |

#### Raw Data For Cycling A.Green


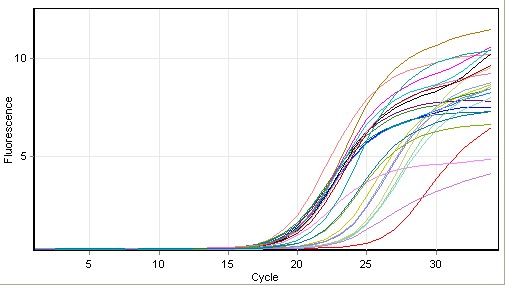


#### Quantitation data for Cycling A.Green


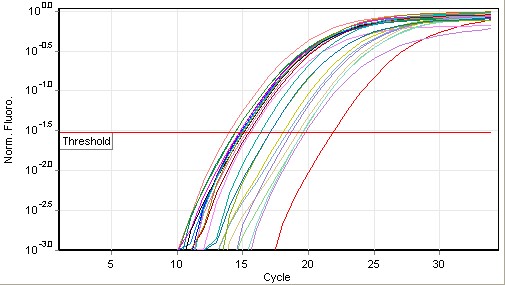


#### Standard Curve


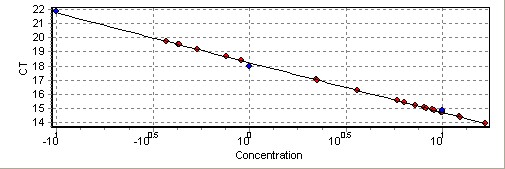


| No. | Colour | Name | Type | Ct | Given Conc (ng/ul) | Calc Conc (ng/ul) | % Var |
| --- | --- | --- | --- | --- | --- | --- | --- |
| 1 | 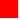 | s0.1 | Standard | 21.86 | 0.1 | 9.26404406896964E-02 | 7.4% |
| 2 | 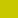 | s1 | Standard | 18.00 | 1 | 1.16519543734212 | 16.5% |
| 3 | 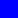 | s10 | Standard | 14.84 | 10 | 9.26404406896964 | 7.4% |
| 4 | 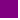 | control | Unknown | 14.75 |  | 9.8317989454753 |  |
| 5 | 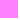 | control | Unknown | 15.55 |  | 5.80978377500951 |  |
| 6 | 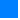 | control | Unknown | 14.88 |  | 9.05520321905945 |  |
| 7 | 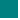 | 6 hour | Unknown | 14.41 |  | 12.3374827799171 |  |
| 8 | 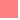 | 6 hour | Unknown | 13.95 |  | 16.6491871377112 |  |
| 9 | 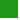 | 6 hour | Unknown | 14.42 |  | 12.2077081208216 |  |
| 10 | 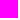 | 1 day | Unknown | 14.74 |  | 9.92647085497306 |  |
| 11 | 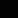 | 1 day | Unknown | 15.05 |  | 8.07419287931069 |  |
| 12 | 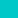 | 1 day | Unknown | 14.91 |  | 8.84230010552813 |  |
| 13 | 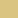 | 3 day | Unknown | 19.18 |  | 0.536681717090584 |  |
| 14 | 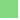 | 3 day | Unknown | 19.53 |  | 0.42824991241535 |  |
| 15 | 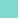 | 3 day | Unknown | 19.52 |  | 0.430947170597693 |  |
| 16 | 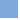 | 5 day | Unknown | 18.38 |  | 0.911223585413378 |  |
| 17 | 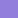 | 5 day | Unknown | 18.65 |  | 0.762555285467739 |  |
| 18 | 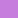 | 5 day | Unknown | 19.74 |  | 0.371646561160242 |  |
| 19 | 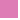 | 3 testosteron | Unknown | 15.23 |  | 7.18520161851961 |  |
| 20 | 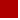 | 3 testosteron | Unknown | 15.41 |  | 6.36247516683649 |  |
| 21 | 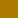 | 3 testosteron | Unknown | 15.03 |  | 8.19786433794116 |  |
| 22 | 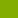 | 5 testosteron | Unknown | 17.03 |  | 2.20849705045007 |  |
| 23 | 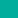 | 5 testosteron | Unknown | 16.28 |  | 3.60041739376707 |  |
| 24 | 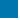 | 5 testosteron | Unknown | 17.01 |  | 2.23714347936885 |  |

**Legend:**
NEG (NTC) - Sample cancelled due to NTC Threshold.
NEG (R. Eff) - Sample cancelled as efficiency less than reaction efficiency threshold.

| This report generated by Rotor-Gene Q Series Software 2.0.2 (Build 4) Copyright 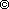2008 Corbett Life Science, a QIAGEN Company. All rights reserved. ISO 9001:2000 (Reg. No. QEC21313) |
| --- |

#### Reaction Efficiency for beta actin

#### Experiment Information

| Run Name | actin gonadektomi |
| --- | --- |
| Run Start | 4/4/2012 11:27:38 AM |
| Run Finish | 4/4/2012 1:42:25 PM |
| Operator | Evelyn |
| Notes |  |
| Run On Software Version | Rotor-Gene 2.0.2.4 |
| Run Signature | The Run Signature is valid. |
| Gain Green | 5. |

#### Quantitation Information

| Threshold | 0.1308 |
| --- | --- |
| Left Threshold | 1.000 |
| Standard Curve Imported | No |
| Standard Curve (1) | conc= 10^(-0.279*CT + 6.360) |
| Standard Curve (2) | CT = -3.582*log(conc) + 22.780 |
| Reaction efficiency (*) | 0.90197 (* = 10^(-1/m) - 1) |
| M | -3.58162 |
| B | 22.78033 |
| R Value | 1.000 |
| R^2 Value | 1.000 |
| Start normalising from cycle | 1 |
| Noise Slope Correction | No |
| No Template Control Threshold | 0% |
| Reaction Efficiency Threshold | Disabled |
| Normalisation Method | Dynamic Tube Normalisation |
| Digital Filter | Light |
| Sample Page | Page 1 |
| Imported Analysis Settings |  |

#### Profile

| Cycle | Cycle Point |
| --- | --- |
| Hold @ 42°c, 10 min 0 secs |  |
| Hold 2 @ 95°c, 5 min 0 secs |  |
| Cycling (34 repeats) | Step 1 @ 95°c, hold 15 secs |
|  | Step 2 @ 60°c, hold 30 secs |
|  | Step 3 @ 72°c, hold 60 secs, acquiring to Cycling A([Green][1][1]) |
| Melt (50-90°c) , hold secs on the 1st step, hold 10 secs on next steps, Melt A([Green][1][1]) |  |
| Hold 3 @ 72°c, 5 min 0 secs |  |
| Hold 4 @ 25°c, 1 min 0 secs |  |

#### Raw Data For Cycling A.Green


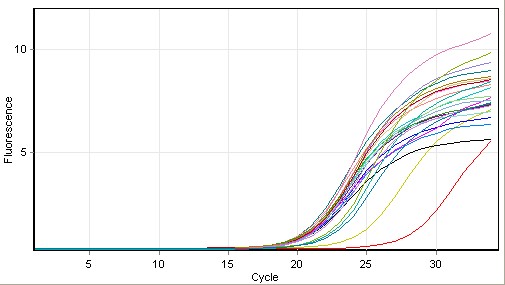


#### Quantitation data for Cycling A.Green


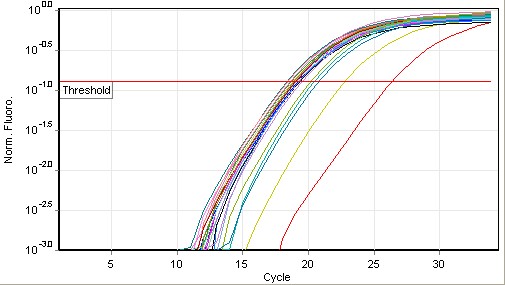


#### Standard Curve


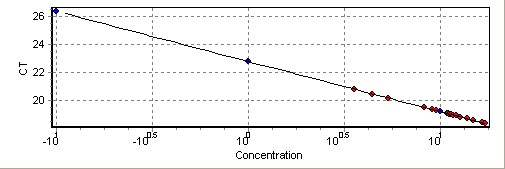


| No. | Colour | Name | Type | Ct | Given Conc (ng/ul) | Calc Conc (ng/ul) | % Var |
| --- | --- | --- | --- | --- | --- | --- | --- |
| 1 | 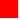 | s0.1 | Standard | 26.36 | 0.1 | 0.100001348845475 | 0.0% |
| 2 | 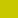 | s1 | Standard | 22.78 | 1 | 0.999973023636303 | 0.0% |
| 3 | 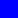 | s10 | Standard | 19.20 | 10 | 10.0001348845475 | 0.0% |
| 4 | 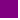 | control | Unknown | 19.06 |  | 10.9444856418491 |  |
| 5 | 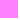 | control | Unknown | 18.69 |  | 13.8339900919964 |  |
| 6 | 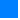 | control | Unknown | 19.28 |  | 9.51459103744985 |  |
| 7 | 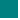 | 6 hour | Unknown | 18.36 |  | 17.1430629336501 |  |
| 8 | 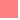 | 6 hour | Unknown | 18.41 |  | 16.5781987268468 |  |
| 9 | 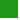 | 6 hour | Unknown | 18.70 |  | 13.7926273198062 |  |
| 10 | 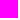 | 1 day | Unknown | 19.02 |  | 11.1853619442677 |  |
| 11 | 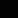 | 1 day | Unknown | 19.36 |  | 9.03651862721014 |  |
| 12 | 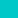 | 1 day | Unknown | 18.91 |  | 12.0441147867955 |  |
| 13 | 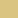 | 3 day | Unknown | 18.82 |  | 12.7286003295273 |  |
| 14 | 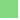 | 3 day | Unknown | 18.97 |  | 11.620897668273 |  |
| 15 | 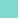 | 3 day | Unknown | 19.04 |  | 11.1041869263411 |  |
| 16 | 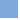 | 5 day | Unknown | 19.28 |  | 9.49997194579711 |  |
| 17 | 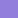 | 5 day | Unknown | 19.06 |  | 10.9214806339451 |  |
| 18 | 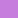 | 5 day | Unknown | 19.50 |  | 8.25746606847227 |  |
| 19 | 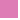 | 3 testosteron | Unknown | 18.59 |  | 14.8167368327344 |  |
| 20 | 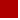 | 3 testosteron | Unknown | 19.00 |  | 11.3568470750208 |  |
| 21 | 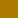 | 3 testosteron | Unknown | 18.90 |  | 12.1125396899009 |  |
| 22 | 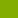 | 5 testosteron | Unknown | 20.18 |  | 5.32792448540437 |  |
| 23 | 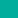 | 5 testosteron | Unknown | 20.46 |  | 4.44239022093719 |  |
| 24 | 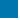 | 5 testosteron | Unknown | 20.81 |  | 3.54564851306571 |  |

**Legend:**
NEG (NTC) - Sample cancelled due to NTC Threshold.
NEG (R. Eff) - Sample cancelled as efficiency less than reaction efficiency threshold.

| This report generated by Rotor-Gene Q Series Software 2.0.2 (Build 4) Copyright 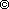2008 Corbett Life Science, a QIAGEN Company. All rights reserved. ISO 9001:2000 (Reg. No. QEC21313) |
| --- |
