# Supplementary material for: Sperm-associated antigen 11A is expressed exclusively in the principal cells of the mouse caput epididymis in an androgen-dependent manner
Source: Reprod Biol Endocrinol. 2013 Jul 1;11:59. doi: 10.1186/1477-7827-11-59 (PMC3710511; doi:10.1186/1477-7827-11-59)
Supplement: Additional file 4: Figure S3 — Analyses of SPAG11A signal peptide using SignalP program. The green line indicates the S score (signal peptide). The peak of the red vertical line (C score) indicates a predicted cleavage site. The blue line (Y score) constitutes a combination of the S and C scores for a better prediction of a cleavage site. A cleavage site is located where the green line meets the highest peak (highest score) of the red line. In SPAG11A, the cleavage site is located at amino acid 20 (D: aspartate). [file 1477-7827-11-59-S4.ppt]

## Slide 1
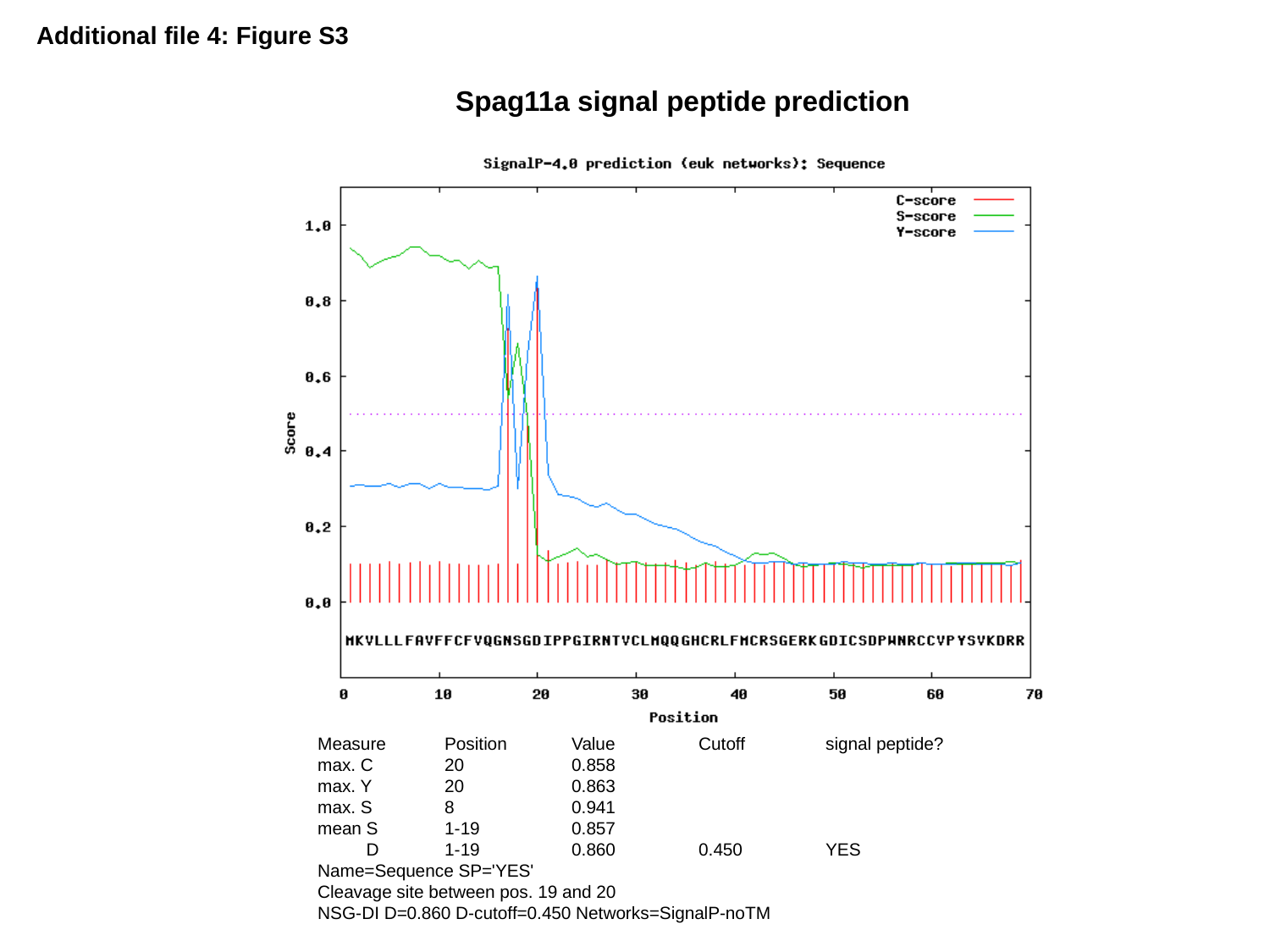

Additional file 4: Figure S3
Spag11a signal peptide prediction
Measure 	Position 	Value 	Cutoff 	signal peptide?
max. C 	20 	0.858
max. Y 	20 	0.863
max. S 	8 	0.941
mean S 	1-19 	0.857
 D 	1-19 	0.860 	0.450 	YES
Name=Sequence SP='YES'
Cleavage site between pos. 19 and 20
NSG-DI D=0.860 D-cutoff=0.450 Networks=SignalP-noTM
